# Nonequilibrium Self-Assembly Control by the Stochastic Landscape Method

**DOI:** 10.1021/acs.jcim.4c02366

**Published:** 2025-04-08

**Authors:** Michael Faran, Gili Bisker

**Affiliations:** † Department of Biomedical Engineering, Faculty of Engineering, Tel Aviv University, Tel Aviv 69978, Israel; ‡ The Center for Physics and Chemistry of Living Systems, Tel Aviv University, Tel Aviv 6997801, Israel; § The Center for Nanoscience and Nanotechnology, Tel Aviv University, Tel Aviv 6997801, Israel; ∥ The Center for Light-Matter Interaction, Tel Aviv University, Tel Aviv 6997801, Israel; ⊥ The Center for Computational Molecular and Materials Science, Tel Aviv University, Tel Aviv 6997801, Israel

## Abstract

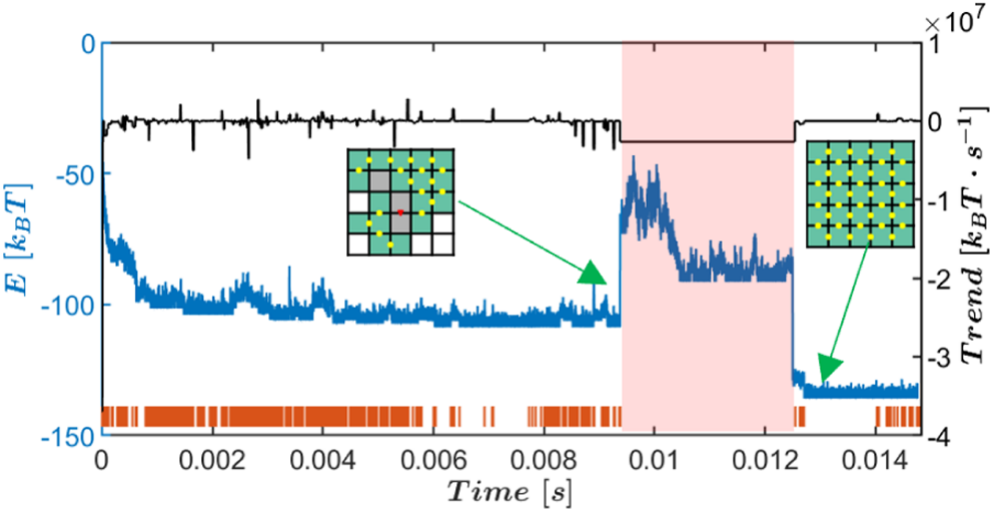

Self-assembly of building blocks is a fundamental process in nanotechnology, materials science, and biological systems, offering pathways to the formation of complex and functional structures through local interactions. However, the lack of effective error correction mechanisms often limits the efficiency and precision of assembly, particularly in systems with strong binding energies. Inspired by cellular processes and stochastic resetting, we present a closed-loop feedback control method that employs transient modulations in interaction energies, mimicking, for instance, the global effect of pH changes as nonequilibrium drives to optimize assembly outcomes in real time. By leveraging the stochastic landscape method, a framework using energy trend-based segmentation to predict self-assembly behavior, our approach dynamically analyzes the system’s state and energy trends to guide control actions. We show that the transient energy modulation during kinetic trapping conditions substantially enhances assembly yields and reduces assembly times across diverse scenarios. This strategy provides a broadly applicable, data-driven framework for optimizing nonequilibrium assembly processes, with potential implications for precision manufacturing and responsive materials design, while also advancing our understanding of controlled molecular assembly in biological and synthetic contexts.

## Introduction

Self-assembly is a fundamental and ubiquitous process observed across physical, chemical, and biological systems.^1−6^ A quantitative understanding of self-assembly is crucial for advancing diverse fields including nanotechnology,^7,8^ materials science,^9^ and computational science.^10,11^ Moreover, it provides insights into key scientific questions, such as the origins of life (Abiogenesis).^12^ At its core, self-assembly involves the emergence of order from initially disordered constituents through local interactions. This process drives the formation of diverse and complex structures from common molecular building blocks within living systems,^13,14^ resulting in a broad range of functional architectures.^8,15−18^ In biological contexts, self-assembly underpins key intracellular processes, including the role of molecular chaperones in protein folding,^19^ chromatin-mediated gene expression regulation,^20^ the spindle-assembly checkpoint during mitosis,^21^ peptide-based supramolecular structures,^22,23^ and the formation of protofilaments in microtubules.^24^

The structural complexity of building blocks gives rise to intricate energy landscapes with numerous local minima, each corresponding to a metastable state.^25−27^ These metastable states often act as kinetic traps, obstructing or delaying the assembly process from reaching the desired configuration.^28−33^ To overcome this challenge, living systems rely on nonequilibrium drive via the utilization of chemical fuel, to transition from “off-target” states to the desired configurations.^34−37^ The added benefits of nonequilibrium driving mechanisms have been widely demonstrated through experimental studies,^38−40^ simulations,^28−30,41−43^ and theoretical models.^44−47^ Biological examples include microtubules leveraging guanosine triphosphate (GTP) hydrolysis for dynamic instability and self-repair,^48^ kinetic proofreading in enzyme–substrate interactions,^49,50^ and adenosine triphosphate (ATP)-driven actin-treadmilling.^51^

Inspired by these biological strategies, researchers have sought to develop efficient control protocols for guiding self-assembly in synthetic and industrial systems.^52−54^ Such protocols aim to replicate the precision of nonequilibrium drives in natural systems, providing a means to navigate energy landscapes and improve assembly efficiency. Mimicking the drives observed in nature allows for greater precision in guiding synthetic self-assembly processes,^28,41,42,54−62^ paving the way for diverse applications, including artificial light-harvesting systems,^63^ viral capsid assembly,^64^ targeted drug delivery,^65^ and supramolecular hydrogels.^27,66^ Despite significant advancements, achieving a comprehensive understanding of nonequilibrium drives in self-assembly remains challenging.^31,57,67,68^

Feedback-based self-assembly control protocols within closed-loop frameworks have shown promise in extending the utility of nonequilibrium drives beyond nanoscale applications.^69−73^ From a modeling perspective, certain nonequilibrium drives can be interpreted as closed-loop control protocols,^5,66,74−80^ where systems identify and respond to specific states to guide assembly processes.^52^ These protocols are particularly effective for systems with high interaction energies, enabling escapes from kinetic traps and progression toward desired configurations.^81^ Various closed-loop control strategies have emerged over time, including dynamic programming,^82,83^ reinforcement learning,^84−86^ and other approaches.^82,87,88^ Combining real-time feedback, such approaches could advance practical applications in synthetic and industrial systems.

Cellular systems often employ stochastic resetting as a control strategy for iterative correct errors and guide processes toward desired outcomes.^89^ For example, chaperonin-mediated protein folding involves the GroEL/GroES complex recognizing and correcting misfolded proteins trapped in stable intermediates.^90^ These chaperonins use ATP-driven cycles to induce transient microenvironmental shifts,^19^ facilitating the escape from kinetic traps. This iterative process, in which repeated cycles occur until the protein reaches its native state, mirrors the concept of stochastic resetting.^19^ Similarly, autophagy employs pH-dependent shocks to reset cellular components, facilitating degradation and overcoming energy barriers.^91^ These biological systems highlight how iterative corrections can effectively navigate complex energy landscapes.

Inspired by these natural strategies, effective closed-loop control protocols aim to maximize assembly yields, minimize assembly times, and meet essential constraints, such as the reliance on a single order parameter,^92−94^ grounding in stochastic models,^95^ integration of data-driven insights with physical models,^83^ explainability,^93,96^ and minimized data and computational demands.^82,93,97^ Despite progress in addressing individual aspects,^86,97^ developing a general nonequilibrium-driven closed-loop strategy for self-assembly that addresses all these challenges remains elusive. A critical prerequisite is identifying the physical framework or parameters upon which such protocols can reliably operate.

The stochastic landscape method (SLM) can provide a robust framework for controlling nonequilibrium self-assembly by enabling macrostate classification.^31,98^ SLM is a recently developed approach^31^ that uses trend-based segmentation of physical parameter trajectories to analyze system dynamics and identify kinetic traps. It applies the Bayesian Estimator of Abrupt Changes (BEAST),^99^ which partitions a time series into segments, each with a distinct trend and noise level. BEAST assumes that a signal consists of noisy segments where the trend—defined as the underlying direction or pattern of change—remains stable within each segment but shifts abruptly at transition points. SLM models this trend as a linear fit and defines segments as the intervals between consecutive transition points.

A key assumption of SLM is that nonequilibrium self-assembly follows transition state theory, meaning the system spends much less time in transition states than in metastable ones. Metastable states are characterized by prolonged dwelling times and near-zero energy trends, where the energy fluctuates around a mean without significant drift in the energy landscape. In contrast, transition states exhibit high trend values and short durations. Each BEAST-identified segment corresponds to a system state, possessing its own steady-state statistics. From these segmented trajectories, SLM extracts stochastic coordinates (energy mean, standard deviation, and trend), coarse-graining the system dynamics into macrostate sequences. It then constructs a predictive map, termed the stochastic landscape, by interpolating a measured physical parameter as a function of these stochastic coordinates.

Previously, this approach has been used to predict self-assembly times based on energy trajectories^31^ for various system conditions, including variations in external driving forces, lattice dimensions, and particle number, ensuring its robustness over a broad parameter space. It can also potentially predict segment dwelling times based on the energy trend stochastic coordinate, aiding in kinetic trap detection. Identifying such traps—characterized by long dwelling times and near-zero trends—can help devise strategies to overcome these barriers and improve assembly efficiency.

Here, we address the challenges of controlling nonequilibrium self-assembly by introducing a closed-loop protocol that leverages the predictive power of the SLM to improve assembly outcomes. Specifically, when energy trends indicate a kinetic trap—identified through the predictive map, referred to as the trap region—the protocol applies shocks of transiently reduced interaction energies, inspired by pH changes. To evaluate this approach, we use a Kinetic Monte Carlo (KMC) toy model^100−102^ to characterize macrostate dynamics under equilibrium conditions, and construct the SLM predictive map for identifying the trap regions. Our closed-loop protocol dynamically monitors energy trends of the BEAST of segments and immediately triggers shocks to allow the system to escape from unfavorable metastable states and optimize the assembly process. We demonstrate that this strategy significantly increases assembly yield and reduces the time to first assembly compared to uncontrolled systems. A nonmonotonic relationship between the amplitude of the drive and the assembly yield emerges, highlighting the critical role of parameter fine-tuning to optimize the assembly efficiency. A detailed analysis of the system’s structural distance from target configurations before, during, and after the shocks reveal the underlying mechanisms that resembles stochastic resetting. Finally, we qualitatively compare the suggested control protocol to other methods. This data-driven framework can be extended to other nonequilibrium processes, with potential applications in precision manufacturing and material design, while advancing our understanding of controlled molecular assembly in synthetic and biological systems.

## Equilibrium Self-Assembly Model

**Figure 1:**
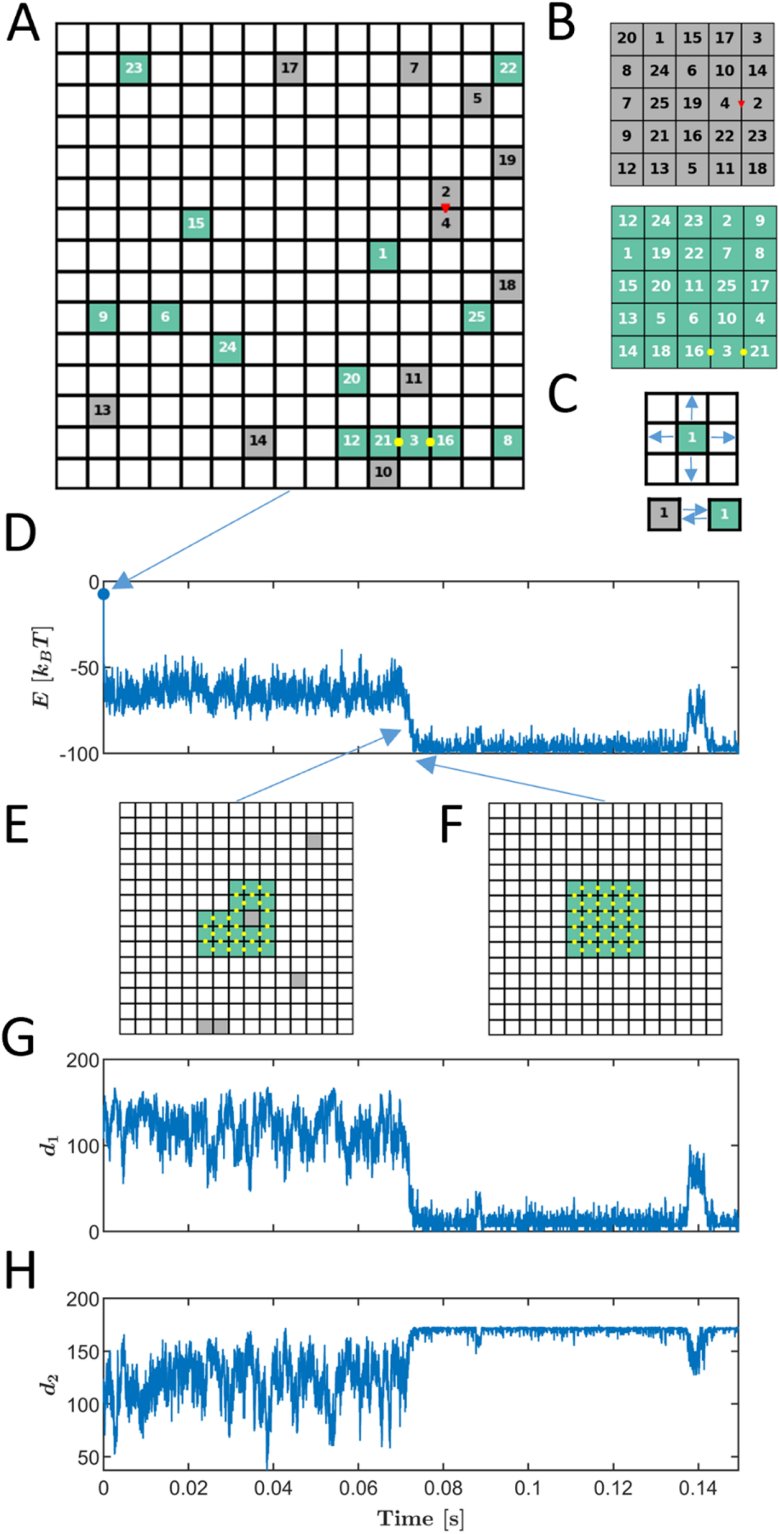
Model System Illustration. (A) A 15 × 15 lattice (*L* = 15) with *N* = 25 distinguishable particles, each in one of *M*_*T*_ = 2 internal states (green or gray), labeled by numbers 1, ···, 25. Red triangles and yellow circles indicate bonds between adjacent particles, corresponding to a specified target configuration. The snapshot represents the system’s initial conditions. (B) Two target structures, each with a distinct spatial arrangement of the *N* particles. (C) Each iteration involves randomly selecting a move from a finite set, which can either be a physical displacement on the lattice or a switch in the internal state. Move probabilities are updated based on the system’s new state after each move. (D) A typical energy trajectory for *J*_*s*_ = −2.5 [*k*_B_*T*], with snapshots (E) showing a near-assembly kinetic trap and (F) showing the system during the first assembly. The distance values from target trajectories are depicted in (G) and (H).

We use the kinetic Monte Carlo (KMC) algorithm,^103^ equivalent to the Gillespie algorithm,^104^ to model nonequilibrium self-assembly with a realistic simulated time scale, improving on previous discrete-time approaches.^30,31^ Our model investigates the self-assembly dynamics of *N* distinguishable particles, each uniquely labeled (*a* = 1, ···, *N*) and characterized by an internal state, *s_a_* = 1, ···, *M*_*T*_, corresponding to a specific target structure, out of the *M*_*T*_ possible encoded targets. This modeling approach draws inspiration from biological systems, where particles adopt different conformations while assembling into predetermined structures. As shown in Figure 1A, the system is initialized with the particles randomly distributed across the grid of an *L* × *L* lattice. Each particle occupies exactly one tile, starting in a random configuration. The target structures, encoded in the interaction energies between the particles, are represented by particular spatial arrangements of all the particles in the system (Figure 1B). The targets are the global minima of the system and are thus the possible goals of the self-assembly process.

The particles interact with their nearest neighbors through pairwise interactions, *J*(*s*_*a*_, *s*_*b*_), which depend on their internal states and the encoded target structures. The interaction promotes strong attractions when the states match within a target structure, while weaker interactions occur for mismatches or between nontarget neighbors. Particle pairs are considered nearest neighbors if they share an edge according to their lattice positions. A pair is classified as “neighboring” if it is designated as nearest neighbors in at least one of the encoded target matrices (Figure 1B). The strength of a pairwise interaction depends on the following conditions:

- Strong attraction (*J*_*s*_): If two nearest neighbors on the lattice both hold the same internal state matching a target configuration (*s*_*a*_ = *s*_*b*_ = *m*) and they are a neighboring pair in the target *m*, they experience a strong, attractive interaction. Also, a strong interaction occurs if the particles are in different internal states (*s*_*a*_ = *m*, *s*_*b*_ = *n*, *m* ≠ *n*), but they are a neighboring pair in both the targets *m* and *n*.
- Weak attraction (*J*_*w*_): If two nearest neighbors on the lattice are not defined as a neighboring pair in any of the targets, or if none of the two particles holds an internal state matching target configurations where they are a neighboring pair, they experience a weak interaction.
- Intermediate attraction : If two nearest neighbors on the lattice are a neighboring pair in stored target *m*, but not on *n* ≠ *m* and attain the states (*s*_*a*_ = *m*, *s*_*b*_ = *n*), they experience intermediate attraction.

Analytically, the interaction of particles *i* and *j* can be rewritten as
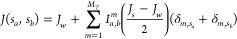
1where *I*_*a*,*b*_^*m*^ is a matrix representing the stored target, *I*_*a*,*b*_^*m*^ = 1 if the particles *a* and *b* are nearest neighbors according to target *m*, and zero otherwise, and δ_*m*,*s*_*a*__ is the Kronecker delta. We now define *B*(*a*, *b*) as the nearest neighbors matrix of the board. *B*(*a*, *b*) attains the value of 1 if particles *a* and *b* are currently nearest neighbors on the lattice and zero otherwise. The total energy of the system *E*, then follows
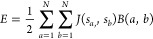
2

At each simulation step, a particle can move to a neighboring tile or undergo a state transition, representing a conformational change (Figure 1C). The set of available moves and their probabilities is updated at each step based on the system’s current state. Using the *n*-fold way KMC algorithm,^103^ we probabilistically select the next move, allowing the system to evolve according to its energy landscape. This approach ensures that the dynamics reflect the assembly process continuously and realistically.

The simulation begins with a random initial state, defined by the position and internal state of each of the *N* particles (an example is depicted in Figure 1A). In each KMC step, the system’s possible moves include 4*N* single-particle translations (representing 4 possible two-dimensional (2D) moves for each particle) and (*M*_*T*_ – 1)*N* state-switch moves (representing the transitions to another internal state), resulting in a total of *N*_tot_ = 4*N* + (*M*_*T*_ – 1)*N* = (3 + *M*_*T*_)*N* possible transitions in a single KMC step. For translations, the corresponding transition rates *r*_*ij*_, from state *j* with energy *E*_*j*_ to state *i* with energy *E*_*i*_, are calculated according to the following energy-dependent formula^100,105−108^
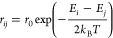
3where the Boltzmann constant is denoted by *k*_B_ and *T* represents the temperature, and all the energy values are expressed in proportion to these parameters. Forbidden translations due to occupied neighbor tiles have *r*_*ij*_ = 0. We chose a rate constant *r*_0_ ≈ 2.5 × 10^6^ s^–1^, to which all the move rates are proportionate. For calculating *r*_0_ in our assembly model, we considered the time required for a particle to diffuse a distance equivalent to its size, corresponding to movement between adjacent tiles in our lattice model. This time scale represents the diffusion of a free particle in the simulation, where the initial and final energies are equal (*E*_*i*_ = *E*_*j*_). Please refer to the rate constant calculation in the Supporting Information (SI) for more details. Similarly, the transition rates *q*_*ij*_ for state-switch moves, relevant when *M*_*T*_ > 1, are
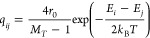
4Note that we simulate a scenario where the state-switch and translation moves occur with a similar likelihood for a specific particle when *E*_*i*_ = *E*_*j*_. Hence, the factor 4*r*_0_ accounts for the four possible translations, while the denominator *M*_*T*_ – 1 reflects the number of potential state flips.

The KMC cumulative rate function used for the selection of the next move and for determining the next state of the system, is calculated according to
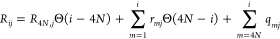
5where Θ represents the Heaviside function. The cumulative rate function is designed such that for *i* > 4*N*, state switch moves are considered as the KMC algorithm move choice, and for *i* ≤ 4*N*, solely translation moves are considered. The total escape rate from a given state *j* is thus *Q* = *R*_*N*_tot_,*j*_. A uniform random number *u* ∈ (0, 1] determines the next state, by identifying the index *i* = *I* such that *R*_*I*–1,*j*_ < *uQ* ≤ *R*_*I*,*j*_. Once the system transitions to the new state, another random number *u*′ ∈ (0, 1] is used to update the time increment by , where *k* is a running index associated with each transition, starting from *k* = 1 up to *k* = *T*_cap_, the total number of KMC steps. The total simulation time is, therefore, *T*_tot_ = ∑_*k*=1_^*T*_cap_^ Δ*t*_*k*_.

Key macro-parameters are recorded throughout the realization, including the system’s total energy (Figure 1D), snapshots of the system along its dynamic trajectory (Figure 1E), and the distance from the stored target configurations (Figure 1G,H). The distance to each target, denoted as *d*_*m*_, is calculated separately for each target by comparing the current adjacency matrix of the particles with the adjacency matrix of the two targets.^31^ Unless stated otherwise, the KMC simulation default parameters follow Table 1.

**Table 1:** Default Simulation Parameters

parameter	notation	value
number of particles	*N*	25
grid size	*L*	15
number of targets	*M*_*T*_	2
strong energy	*J*_*s*_	–2.5 [*k*_B_*T*]
weak energy	*J*_*w*_	–1 [*k*_B_*T*]
simulation time cap	*T*_cap_	5 × 10^7^

## Results and Discussion

### Equilibrium Inference

To evaluate the self-assembly process under equilibrium conditions, we ran the kinetic Monte Carlo (KMC) simulations with the parameters listed in Table 1, scanning across a range of the strong interaction values *J*_*s*_ = −3.6, ···, −2 [*k*_B_*T*]. For each realization, we record the time to the first assembly, *T*_FAS_, defined as the time required for the system to reach a complete target structure for the first time during a simulation. In cases where no assembly events occurred during the simulation, *T*_FAS_ was taken to be the simulation length *T*_tot_. These *T*_FAS_ results are collected and referred to as the unassembled group.

**Figure 2:**
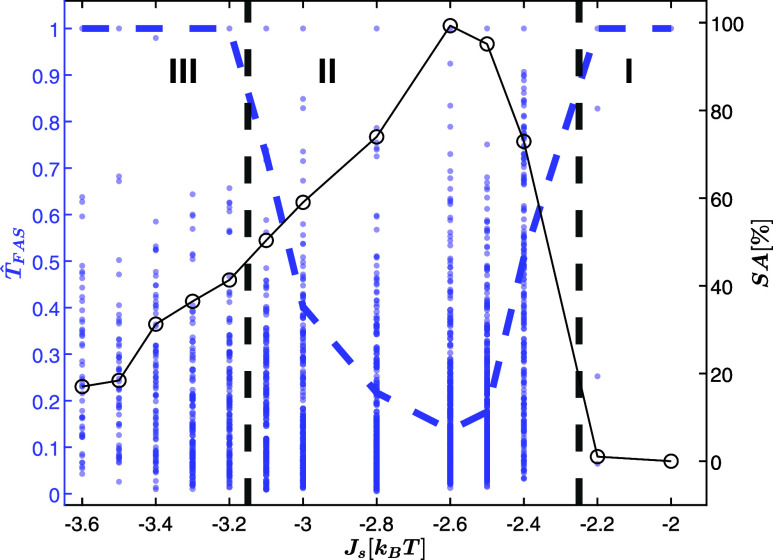
Equilibrium simulation results for the parameters in Table 1, across a range of *J*_*s*_ values. The left *y*-axis shows the normalized *T̂*_FAS_ distribution versus *J*_*s*_, based on 288 simulation realizations. *T̂*_FAS_ is the assembly time, *T*_FAS_, normalized by the maximum simulation time per *J*_*s*_. The dashed purple line represents the median *T̂*_FAS_(*J*_*s*_). Pale purple dots indicate values where assemblies occurred within the simulation time frame. Nonassembled results are grouped at *T̂*_FAS_ = 1. The black line corresponds to the right *y*-axis. It shows the assembly percentage versus *J*_*s*_. Dashed vertical black lines qualitatively separate the three self-assembly regions (I–III) discussed in the text.

The consistency of our simulations was verified by ensuring that *T*_FAS_ as a function of *J*_*s*_ exhibited a convex shape (Figure 2), as predicted in previous studies.^30,31,67,109−116^ The optimal value of *J*_*s*_, which minimizes *T*_*FAS*_, represents the most efficient assembly conditions in equilibrium. The curve of *T*_FAS_ vs *J*_*s*_ is divided into three distinct physical regions: I, the unstable nucleation seed region, II, the efficient equilibrium self-assembly region, and III, the kinetic stagnation region, where kinetic traps are abundant. In regions I and III, assemblies are either rare or unlikely, resulting in high *T*_FAS_ values relative to the total KMC simulation steps *T*_cap_, whereas region II is characterized by lower *T*_FAS_ values.

Figure 2 illustrates the distribution of *T*_FAS_ across the examined *J*_*s*_ values, derived from 288 simulation runs for each energy value. For every *J*_*s*_, the first assembly time *T*_FAS_ was recorded and divided by its maximum across all realizations, resulting in a normalized value *T̂*_FAS_, ranging from 0 to 1. The unassembled group appears as a single point in Figure 2, representing the maximum value of this group after normalization. The median *T̂*_FAS_ was calculated for each *J*_*s*_, demonstrating the convex behavior of *T̂*_FAS_(*J*_*s*_). To observe *T*_FAS_(*J*_*s*_), without the values normalization, see Figure S1.

Moreover, the total assembly yield SA[%] was defined as the relative percentage of simulation realizations in which an assembly event occurred. Three distinct self-assembly regions were identified, with dashed lines marking boundaries between regions I and II at *J*_*s*_ = −3.15 [*k*_B_*T*] and between regions II and III *J*_s_ = −2.25 [*k*_B_*T*]. As expected, region I has low assembly yields of up to 1%, where maximum assembly yields appear for region II, reaching up to 99%. Region III shows assembly yields of 17–41% due to kinetic trapping that impedes the assembly process, suggesting that a control protocol that accelerates the escape from these traps could significantly improve the assembly yield. The optimal value for *J*_*s*_, corresponding to the minimal assembly times, is found at *J*_*s*_ = −2.5 [*k*_B_*T*].

Examples of simulation realizations and their corresponding dynamics, including the total energy *E* and the distances from targets, *d*_1_ and *d*_2_, are shown in the Supporting Movies S1, S2, and S3, for the three distinct regions, *J*_*s*_ = −2.2, −2.5, and −3.5 [*k*_B_*T*], respectively. Similar behaviors of *T̂*_FAS_ vs *J*_*s*_ was also observed for additional cases, including systems of *M*_*T*_ = 3 and *M*_*T*_ = 4 with *N* = 25 particles (Figure S2), as well as for *M*_*T*_ = 2 with *N* = 36 particles (Figure S3).

### Closed Loop Control Drive Protocol

This work aims to improve the assembly time and success rate of self-assembly processes hindered by kinetic traps (as observed in Region III in Figure 2). The nonequilibrium protocol employs interaction energy modulation, inspired by pH changes, within a closed-loop framework. We emphasize that the protocol monitors the system’s energy trend in near real-time, estimated by the SLM at discrete intervals. If the system is suspected to be kinetically trapped, a nonequilibrium drive is conditionally activated in real time based on the measured value. By transiently disrupting unwanted metastable states, this approach effectively kicks the system out of kinetic traps, enabling more efficient and reliable assembly outcomes. Specifically, the shock is implemented as a reduction in interaction energies between assembly constituents,^117^ which is mathematically formulated as new values for the strong and weak binding energies,  and . Our approach assumes that external physical parameters, such as pH or temperature, modify all interaction forces in the system uniformly. In systems where external changes induce additional nonequilibrium effects—such as active molecular chaperones in protein folding—further modifications to the model would be necessary to capture system-specific responses.

**Figure 3:**
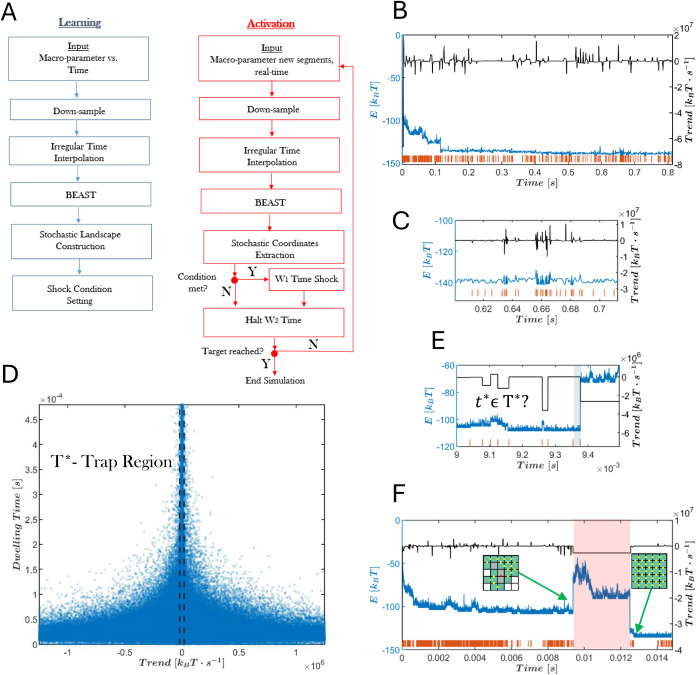
(A) Left: flowchart of the learning phase, Right: flowchart of the drive activation. (B) The total energy versus simulation time (left *y*-axis) for *J*_*s*_ = −3.5 [*k*_B_*T*], with vertical orange lines, indicate trend change points detected by the BEAST in almost real time. The right *y*-axis shows the segment-wise energy trend. (C) A zoomed-in view of the energy, segments, and trend values. (D) The dwelling time versus trend of the overall stochastic landscape segments obtained using the BEAST algorithm in almost real-time. The kinetic trap region (T*), centered around trend zero, defines the conditions for drive activation. (E) A zoomed-in example trajectory of energy, trend, and segments, showing closed-loop drive activation. The shaded blue rectangle marks the first segment when the trend t* is found within region T*, triggering the drive. (F) The energy trajectory before and after drive activation, with the drive period shaded in red. The target is reached after the drive, and the system state is shown schematically before and after activation.

This modification affects the total energy of the system (see eqs 1 and 2), and subsequently, the rates of possible moves (eqs 3 and 4). The altered interactions persist for a duration, *W*_1_, after which equilibrium energy values are restored. Every *W*_2_ seconds, the nonequilibrium drive is conditionally triggered in real-time based on the system’s estimated macro-state, defined by segments of the energy trend trajectory.^31^ Specifically, the drive is activated when the energy trend dynamically indicates the system is trapped. This is determined by whether the current energy trend is found within the prelearned range of values, referred to as the trap region, assessed during the simulation. The learning process of defining the trap region, the BEAST potential activation time-scale *W*_2_, and the shock time *W*_1_ is depicted in Figure 3A left flowchart, while the real-time activation protocol of the drive is shown in Figure 3A right flowchart.

The learning phase consists of several key steps. First, we analyze the resulting *T*_FAS_ as a function of *J*_*s*_ at equilibrium (Figures 2, S2, and S3). For each *J*_*s*_, the BEAST activation window is set as *W*_2_(*J*_*s*_) = 0.01 × median(*T*_FAS_(*J*_*s*_)), making the system monitoring almost real-time, compared to the time to first assembly. An ensemble of total energy trajectories is then simulated under equilibrium conditions, with the BEAST algorithm triggered every *W*_2_ seconds to analyze partial trajectories, which span from either the last detected trend changepoint or from the beginning of the simulation in the case of the first segment. The partial energy trajectory is downsampled and interpolated onto a uniform time vector for computational efficiency.

The BEAST algorithm is then activated for detecting changepoints within the interpolated trajectory, and for calculating linear energy trends for each segment^99^ between consecutive changepoints or between the last changepoint and the current simulation time. This process continues until the maximum allowed KMC steps *T*_cap_ is reached (Figure 3B,C). The segments and their corresponding trends and times are saved for later analysis. Examples of the total energy trajectories, corresponding segments, and trends for *J*_*s*_ = −3.5 [*k*_B_*T*] are shown in Figure S4 in the Supporting Information.

Upon completion of the simulation ensemble, the trends and dwelling times of all the ensemble segments are used for constructing a stochastic landscape^31^ of the dwelling time versus the trend. The trap region T* is defined as the range around zero trend encompassing 20% of the segments (Figure 3D), determined via an iterative expansion around trend zero and binary search to ensure convergence to a region containing between 20 and 21% of the segment points. In the activation phase, the shock is to be triggered when the trend value of the most recently detected segment, abbreviated as t*, evaluated at times of integer multiples of *W*_2_, falls within the trap region T*, manifesting as a reduction in interaction energies. This completes the learning phase and sets the stage for employing the feedback control drive protocol.

With the learning phase complete and the shock condition defined, the feedback control drive protocol can be activated. For each *J*_*s*_ value in the tested range, a new ensemble of total energy trajectories is generated. As in the learning phase, the system’s energy is monitored every interval of *W*_2_ by downsampling the partial energy trajectory, interpolating it onto a uniform time vector for computational efficiency, and segmenting it using the BEAST algorithm. At each interval, the trend of the most recent segment, t*, is evaluated against the shock condition, as illustrated in Figure 3E. If the condition is met, a shock is applied to the system, reducing interaction energies by a factor of ρ, a tuning parameter of the drive. The shock persists for  seconds.

We hypothesize this choice of *W*_1_ is sufficiently long to induce a meaningful perturbation that helps the system escape kinetic traps, yet it remains short relative to the total trajectory time (since *W*_1_ < 0.01 × median(*T*_FAS_(*J*_*s*_))), allowing for multiple drive activations. The gap of  is chosen as a heuristic guess, allowing the system time to relax into a new metastable state after the drive activation, before the next shock is applied. Fine-tuning the shock amplitude ensures that assembly seeds trapped in kinetic traps are reconfigured with high probability. The shock induces a partial disassembly, enabling the system to escape the trap and explore alternative assembly pathways toward global minima associated with the target structures (see Figure 3F).

### Closed Loop Control Drive Activation

The control protocol described in Figure 3 was implemented using the default simulation parameters listed in Table 1, with strong energy values of *J*_*s*_ = −3.4, −3.5, and −3.6 [*k*_B_*T*]. During the learning phase (Figure 3A, left panel), the input data comprised 288 total energy vs time trajectories generated from an ensemble of simulations with identical parameters. For the three *J*_*s*_ values, the relationship between dwelling times and trend values exhibited a consistent pattern, showing a consistent increase in dwelling times as trend values approached zero, regardless of whether the trend was positive or negative, as seen in Figure 3D for *J*_*s*_ = −3.5 [*k*_B_*T*] and in Figures S5 and S6 for *J*_*s*_ = −3.4 [*k*_B_*T*] and *J*_*s*_ = −3.6 [*k*_B_*T*], respectively. Accordingly, unique trap regions T* were defined for each *J*_*s*_ value. We note here that Figures 3D, S5, and S6, display well-defined, densely populated data patterns. Based on this, we hypothesize that the learning phase could have identified similar trap zones using a smaller set of trajectories (fewer than 288). This suggests a potential advantage for other systems, where data collection is limited.

**Figure 4:**
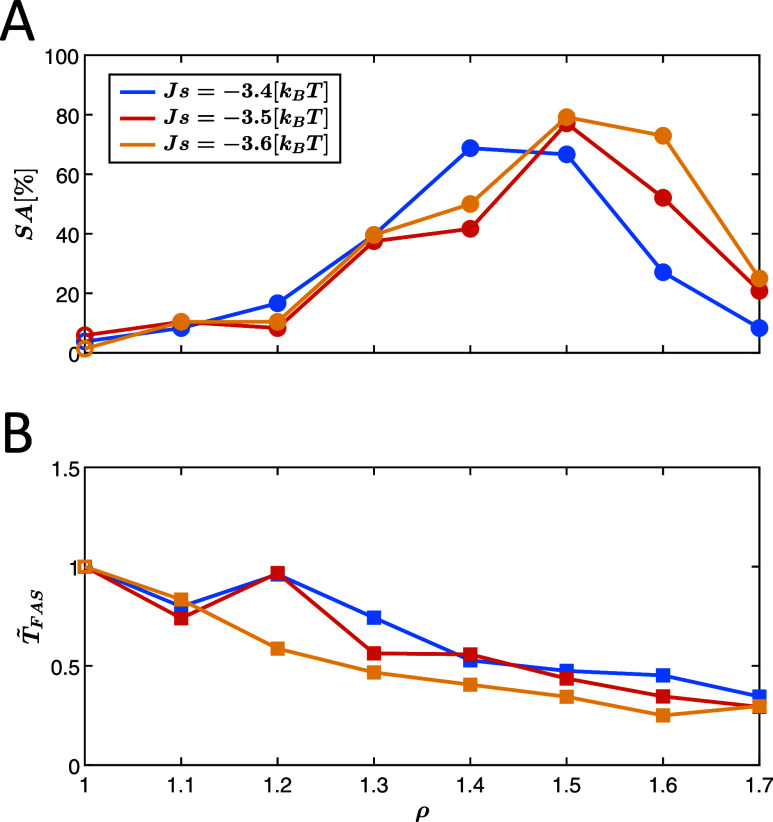
Impact of drive activation on the assembly yield (SA[%]) and the rescaled mean time to first assembly, *T̃*_FAS_, derived by dividing *T̅*_FAS_ by the equilibrium result constant of *T̅*_FAS_(ρ = 1). (A) Assembly yield versus the drive amplitude ρ. (B) Mean rescaled time to first assembly, plotted against the drive amplitude ρ. Results are provided for *J*_*s*_ = −3.4, −3.5, and −3.6 [*k*_B_*T*]. The mean time to first assembly and assembly yield are calculated based on 48 simulation realizations. The empty markers in ρ = 1 depict the equilibrium values.

Subsequently, the drive activation phase (Figure 3A, right panel) was implemented for 48 new trajectories with drive amplitude values of ρ = (1.1, 1.2, ···, 1.7). For each drive amplitude, two key metrics were collected, namely, the mean time to first assembly *T̅*_FAS_, calculated as the average over *T̅*_FAS_ results only for cases where assembly occurred, and the total assembly yield SA[%]. We also calculate *T̃*_FAS_(ρ), the rescaled mean time to first assembly, by dividing *T̅*_FAS_ for each ρ by the uncontrolled (equilibrium value) constant of *T̅*_FAS_(ρ = 1). These results, along with the equilibrium values for these two metrics, are depicted in Figure 4. Notably, for all examined *J*_*s*_ values, there is an optimal drive amplitude, i.e., an energy reduction factor, that corresponds to the peak of the SA(ρ) curve, that significantly enhances assembly yield. Specifically, assembly yields increased from equilibrium values of 31.3, 18.4, and 17, to 98, 91.7, and 95.8%, for *J*_*s*_ = −3.4, −3.5, and −3.6 [*k*_B_*T*], respectively. The optimal drive value for all three energies was found to be ρ = 1.5, corresponding to effective reduced strong and weak binding energy values of *J*_*s,C*_ = −2.26, −2.33, and −2.4 [*k*_B_*T*] and *J*_*w,C*_ = −0.67 [*k*_B_*T*].

To further validate the proposed control strategy, we evaluated whether *T̃*_FAS_ decreased in the successfully assembled simulations. Indeed, as shown in Figure 4B, *T̃*_FAS_ decreased by slightly more than 2-fold for all examined *J*_*s*_ values at ρ = 1.5, further demonstrating the effectiveness of the control protocol. Detailed *T*_FAS_ distributions for equilibrium and nonequilibrium conditions are shown in Figure S7 in the Supporting Information.

To evaluate the robustness of these findings, we repeated the drive activation scheme shown in Figure 3 for *M*_*T*_ = 3 and 4 stored targets with *N* = 25 particles, and for *M*_*T*_ = 2 stored targets with *N* = 36 particles. The results, presented in Figures S8–S10 in the Supporting Information, respectively, demonstrate significant improvements in both assembly yield and *T̃*_FAS_ compared to equilibrium conditions. For optimal ρ values corresponding to maximum yield, assembly yields increased from 20–32 to 85–89, 22.5–41 to 79%, and from 1.4–5 to 69–79%, respectively. The *T̃*_FAS_ values also showed improvements compared to the corresponding equilibrium conditions, showing a reduction of 26–41, 16–19, and 57–66%, respectively.

The curve in Figure 4A reveals a nonmonotonic relationship between the assembly yield and ρ. Specifically, the yield increases with drive amplitude, reaching a maximum at ρ = 1.5 for all examined *J*_*s*_ values, before decreasing for higher amplitudes. In parallel, Figure 4B demonstrates that *T̃*_FAS_ generally decreases with increasing drive amplitude, reaching a minimum at ρ = 1.5 for *J*_*s*_ = −3.4 [*k*_B_*T*] and −3.5 [*k*_B_*T*], *at ρ* = 1.6 for *J*_*s*_ = −3.6 [*k*_B_*T*], before slightly rises at ρ = 1.7.

To understand the observed convexity, we analyzed the drive’s effect on the distance from targets, defined as the minimum distance to any stored targets at a given time. For this purpose, we introduce three average distance metrics: ⟨*d*⟩_before_, ⟨*d*⟩_during_, and ⟨*d*⟩_after_. These correspond, respectively, to average distances upon segments immediately preceding drive activation, during activation, and in the segment preceding the subsequent activation or final assembly. For simulations in which an assembly event occurred, only the segments preceding the first assembly event were included in the analysis. All segments were collected for this analysis in simulations where no assembly event occurred. This approach ensures that the analysis reflects the drive’s longer-term effects, avoiding transitional states where the system has not yet fully relaxed after activation.

**Figure 5:**
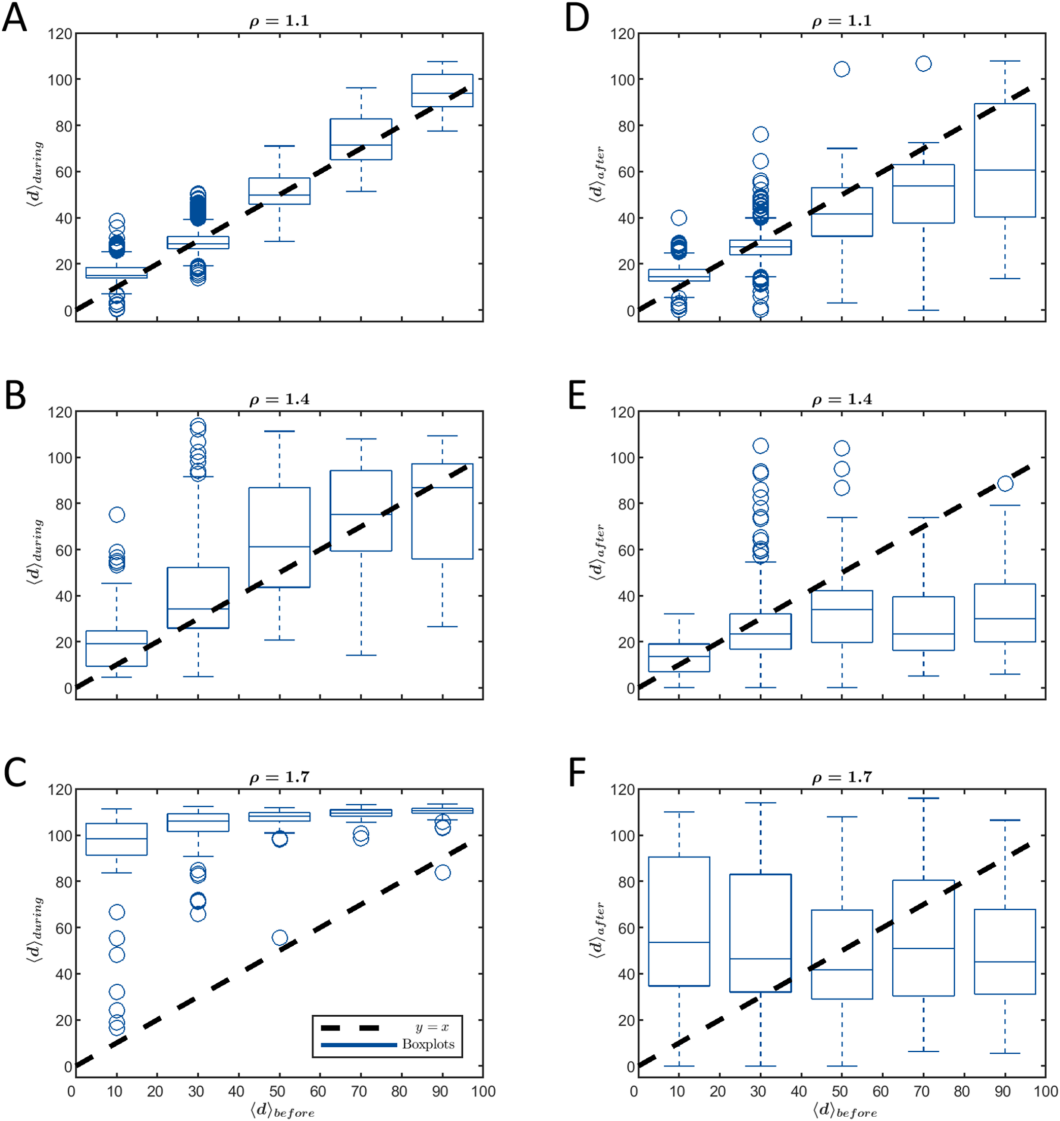
Segments average distance from the targets before, ⟨*d*⟩_before_, during, ⟨*d*⟩_during_, and after, ⟨*d*⟩_after_, drive activation. The left column (A–C) displays ⟨*d*⟩_during_ versus ⟨*d*⟩_before_, while the right column (D–F) illustrates ⟨*d*⟩_after_ versus ⟨*d*⟩_before_ for drive amplitudes of ρ = 1.1, 1.4, and 1.7, respectively. The black dashed line indicates the *y* = *x* line for visual comparison. The boxplots represent the data distribution for each abscissa bin, with bins of 20 distance units widths with centers at 10, 30, 50, 70, and 90. The whiskers denote the standard interquartile range (IQR), and outliers, represented as empty circle markers, are defined as values exceeding 1.5 times the IQR.

As shown in Figure 4A, assembly yields were consistently low at both ρ = 1.1 (low drive amplitude) and ρ = 1.7 (high amplitude), while relatively high yields were observed at ρ = 1.4. For these drive values, the left column of Figure 5 displays ⟨*d*⟩_during_ versus ⟨*d*⟩_before_, while the right column presents ⟨*d*⟩_after_ versus ⟨*d*⟩_before_. Statistical data are visualized using boxplots with data grouped into five data bins, collecting all the averaged segments’ distance data within ± 10 from the centers of 10, 30, 50, 70, and 90 distance values, respectively.

We hypothesize that the optimal ρ, leading to a significant yield increase, would result in slightly larger values of ⟨*d*⟩_during_ relative to ⟨*d*⟩_before_, reflecting the escape of the system from kinetic traps. Such a behavior resembles a partial stochastic resetting where the system does not revert to its initial conditions, but instead, only a subset of the bonds are broken to facilitate successful assembly.

Indeed, for drive amplitude too low (ρ = 1.1, Figure 5A), ⟨*d*⟩_during_ closely aligns with ⟨*d*⟩_before_, suggesting that the system is hardly perturbed by the drive. For the intermediate value (ρ = 1.4, Figure 5B), ⟨*d*⟩_during_ values are slightly higher than ⟨*d*⟩_before_, indicating that the system is “kicked” out of the unwanted trap state, thereby facilitating efficient assembly. Conversely, a drive amplitude too large (ρ = 1.7, Figure 5C) causes significant resetting of the system’s configuration, as evident from the values of ⟨*d*⟩_during_, which ultimately delays the assembly. These values are comparable to the initial condition distance mean (average of 101.6, calculated over 92 trajectories), and in some cases, even exceed it, even when compared against different ⟨*d*⟩_before_ values.

Complementary observations from ⟨*d*⟩_after_ versus ⟨*d*⟩_before_ provide insight into the postdrive progress toward the target. For ρ = 1.1 (Figure 5D), ⟨*d*⟩_after_ slightly falls below ⟨*d*⟩_before_, indicating slow postactivation assembly progress. At ρ = 1.4 (Figure 5E), a pronounced decrease of ⟨*d*⟩_after_ compared to ⟨*d*⟩_before_ reflects significant advancement toward the target. In contrast, for ρ = 1.7 (Figure 5F), ⟨*d*⟩_after_ values show mixed behavior, where high initial distances ⟨*d*⟩_before_ lead to a decrease in ⟨*d*⟩_after_, while low initial distances exhibit the opposite trend of increase in ⟨*d*⟩_after_, suggesting the system resets away from the target.

Further examination of the right column in Figure 5 highlights the superior performance of ρ = 1.4 compared to the other drive values. The extended bottom whisker of the leftmost boxplot, which corresponds to low ⟨*d*⟩_before_ values, reaches zero distance *d*_after_ = 0 (the target) for ρ = 1.4 and ρ = 1.7. However, the *d*_after_ range for ρ = 1.7 is much larger compared to ρ = 1.4, indicating that many realizations are kicked far from the target even if the distance to the target was small before the activation of the drive. In the case of ρ = 1.1, the value *d*_after_ = 0 appears only as outliers. These results highlight that ρ = 1.4 achieves the most consistent proximity to the target among the three drive amplitudes.

In Figure 5E (ρ = 1.4), the median of the leftmost boxplot closely aligns with the dashed line representing *y* = *x*. This suggests that the drive activation at low *d*_before_ values does not lead to a consistent tendency toward higher or lower *d*_after_ values. Nevertheless, the observed increase in the assembly yield for ρ = 1.4 implies that this undirected *d*_after_ response may play a role in the improvement in yield. This improvement could be analogous to a first-passage time problem with uniform resetting,^118^ where the probability of reaching a target within a specific time frame is maximized by tuning the resetting rate to the system’s physical parameters, even in the absence of explicit directional guidance to the target. Further investigation, especially in light of recent studies on stochastic resetting,^119,120^ is necessary to determine the possible contribution of such mechanisms. For *d*_before_ > 50, ρ = 1.4 yields the lowest ⟨*d*⟩_after_ values compared to ρ = 1.1 and ρ = 1.7, suggesting a convex ⟨*d*⟩_after_(ρ) pattern and highlighting the relative advantage of ρ = 1.4. The combined effects of ⟨*d*⟩_after_ reduction at high ⟨*d*⟩_before_ values and the resetting mechanism at low ⟨*d*⟩_before_ values result in an improved assembly yield at ρ = 1.4, compared to low (ρ = 1.1) and high (ρ = 1.7) drive amplitudes.

Exemplary snapshots of board states before and after a drive activation are presented in Figure S11 in the Supporting Information for ρ = 1.1, 1.4, and ρ = 1.7, illustrating the distinct effects of different drive amplitudes. Additionally, Movie S4 demonstrates the simulation dynamics under repeated closed-loop drive activation with ρ = 1.4. This video displays the total energy and distance from the targets for *J*_*s*_ = −3.5 [*k*_B_*T*], allowing qualitative comparison to the equilibrium dynamics video (Movie S3). Drive activation periods are highlighted in pale red, showing how the protocol reduces the distance from the target at high distances, while partially resetting the system at low distances, without showing a clear tendency toward either higher or lower distance values after activation. These examples illustrate how the proposed protocol successfully enables assembly by steering the system toward the target.

Figures 4 and 5 results highlight the importance of carefully tuning the strength of the nonequilibrium modulation to effectively promote escape from kinetic traps, or error correction, without excessively resetting progress toward assembly. This behavior is reminiscent of recent observations by Niblo et al.,^94^ who found that in oscillatory protocols applied to viral capsid assembly binding energies, the amplitude and period of interaction energy oscillations must be carefully matched to the system’s intrinsic relaxation time scales to maximize yield.

### Comparison to Alternative Control Strategies

Several alternative control strategies have been applied to or proposed for self-assembly systems, each with distinct advantages and limitations compared to the present SLM approach, which is designed for small, stochastic systems with irregular kinetic trapping. Additionally, it requires a minimal data set (less than 300 equilibrium trajectories) and has a clear physical interpretability. All comparisons of computational cost, data requirements, and generalizability to other systems in the following paragraphs are made relative to the present SLM approach.

Reinforcement learning (RL) is potentially generalizable to other self-assembly systems.^86,121^ However, RL can require significantly more training data than the SLM and demands carefully designed reward functions, which may be difficult to construct, particularly in sparse-reward self-assembly landscapes.^84^ RL-derived policies can lack clear physical interpretability, limiting mechanistic self-assembly possible insights.

Neuroevolutionary algorithms evolve time-dependent control sequences without requiring predefined system models, making them highly generalizable.^84,122^ While this approach performed well for large (500-particle) systems, its performance may decline in smaller, highly stochastic assemblies when relying on only a single trajectory to evaluate each genome. This could be mitigated by selecting genomes based on ensemble averages, though at increased computational cost. Like RL, neuroevolutionary algorithms may provide limited physical interpretability.

Markov State Model (MSM)-based optimal control optimizes assembly yield once a suitable state space is defined,^28^ a promising generalizable result compared to the SLM. However, extensive unbiased and biased simulation data is required to estimate transition rates, and it is sensitive to the states’ coarse-graining. The SLM avoids these challenges by relying solely on real-time energy trends.

Predefined oscillatory drives provide a simple open-loop alternative.^94^ This approach has been shown to work when a preliminary analysis identifies a specific system’s time scales, though its generalizability remains unclear. The oscillatory binding energy applies a fixed, deterministic schedule. In contrast, the SLM conditionally activates a drive only when a kinetic trap is detected. This conditional triggering also minimizes the total protocol intervention time, potentially reducing costly disruption. In systems displaying temporally irregular kinetic trap patterns across trajectories, periodic drives may underperform relative to the SLM’s adaptive response.

Parallel tempering (PT),^123^ used to enhance sampling in rough energy landscapes, could, in principle, be adapted into an open-loop control, where temperature modulation accelerates assembly. However, compared to the SLM, PT would require careful tuning, multiple parallel replicas, and higher computational cost. Moreover, its experimental feasibility is limited. Such a protocol may be disadvantageous close to the target, when kinetic traps with mean energy similar to the target are abundant. The latter might occur since replica exchange moves are conducted based on energy differences.

Other techniques such as simulated annealing,^124^ dynamic programming,^82,83^ automatic differentiation,^54^ and other approaches^87,88^ could also be compared to the SLM. Overall, these comparisons highlight the SLM’s suitability for small, stochastic systems with heterogeneous trapping behavior. Future benchmarking could clarify the performance and trade-offs of these methods when applied to common self-assembly testbeds.

## Conclusions

We tackled the challenge of developing a comprehensive closed-loop protocol for self-assembly systems that integrates explainability, physical-based modeling, and single-order-parameter control. By leveraging segmented dynamical analysis of energy trajectories, our approach improves self-assembly outcomes by identifying kinetic traps—characterized by high dwelling times and low energy trends—to enable targeted activation of a nonequilibrium drive. This drive transiently reduces the binding energy between system constituents, effectively disrupting unwanted, metastable, kinetically trapped configurations, and facilitating assembly.

The nonequilibrium driving mechanism efficiently improved both first assembly times and assembly yields within the closed-loop protocol. Testing across several self-assembly systems with varying numbers of encoded targets and system sizes revealed significant improvements. Specifically, assembly yields increased from 1.4–41% under equilibrium conditions to 69–98% with the drive, and the time to first assembly decreased by 16–66% compared to equilibrium settings. These improvements were facilitated by the closed-loop feedback control protocol that integrates segmented dynamical analysis of energy trajectories, insights into the relationship between energy trends and segment dwelling times, and nonequilibrium shocks that resulted in partial stochastic resetting.

Our findings suggest that the optimal drive amplitude enhances assembly yield through two distinct but complementary mechanisms. When the system is far from the target configuration, the drive improves proximity to the target by promoting transitions into more favorable configurations postactivation. Near the target, the drive facilitates progress through repeated partial resettings, which lack directional preference but statistically guide the system closer to the target over time. Therefore, only when the system is near the target, can this behavior be intuitively understood as a series of repeated trials in which the system attempts to complete assembly, where the probability of success increases with the number of attempts. Together, these mechanisms highlight the effectiveness of the nonequilibrium drive in overcoming kinetic traps and optimizing self-assembly outcomes. Nonetheless, further investigation is necessary to fully elucidate these dynamics and their role in controlling self-assembly, particularly in the context of stochastic resetting.^119,120^

The control method builds upon the stochastic landscape method (SLM),^31^ which relies on segmenting complex process observables based on trend changes, and the corresponding statistical moments of the resulting segment. The improvements in self-assembly outcomes presented here further validate the SLM as a versatile framework for understanding and controlling complex stochastic systems, providing a quantitative foundation for guiding stochastic processes.

From a broader perspective, the suggested framework is generalizable for broad applications across diverse systems. We hypothesize that this approach is particularly well-suited to systems with energy landscapes rich in local minima, where transitions are governed by Kramers’ escape rate.^125^ The proposed drive protocol could be extended to other self-assembly systems via numerical simulations, incorporating virtual Monte Carlo move algorithms for physical cluster motion.^108,126^ Moreover, combining our approach with state-of-the-art self-assembly tracking and inference techniques^127^ may enable the creation of an end-to-end control protocol for realistic experimental applications. Potential interest also lies in systematically benchmarking our approach against a broader class of closed-loop and open-loop strategies. Future work could quantitatively evaluate these approaches under common conditions to further clarify their relative performance and applicability across different self-assembling systems.

In summary, our method offers a framework for advancing closed-loop nonequilibrium control of self-assembly processes. Beyond its immediate applications in self-assembly systems, this framework offers a generalizable approach for guiding stochastic processes in systems characterized by complex energy landscapes, paving the way for applications in experimental settings.

## Data Availability

The data and scripts used to generate the results and figures in this study, including those in the main text and Supporting Information, are publicly available at: https://github.com/michaelfaran/Nonequilibrium-Self-Assembly-Control-by-the-Stochastic-Landscape-Method/tree/main.

## Supporting Information Available

Simulation realization for equilibrium for *J*_*s*_ = −2.2 [*K*_B_*T*] (AVI)
Simulation realization for equilibrium for *J*_*s*_ = −2.5 [*K*_B_*T*] (AVI)
Simulation realization for equilibrium for *J*_*s*_ = −3.5 [*K*_B_*T*] (AVI)
Simulation realization under nonequilibrium drive with ρ = 1.4 for *J*_*s*_ = −3.5 [*K*_B_*T*] (AVI)
Rate constant calculation for the KMC, additional nonequilibrium simulation results, and additional equilibrium simulation results; equilibrium simulation results for *M*_*T*_ = 2, *N* = 25 without normalization (Figure S1); equilibrium simulation results for *M*_*T*_ = 3, *N* = 25 and *M*_*T*_ = 4, *N* = 25 (Figure S2); equilibrium simulation results for *M*_*T*_ = 2, *N* = 36 (Figure S3); typical energy trajectories vs time for equilibrium with *J*_*s*_ = −3.5 [*K*_B_*T*] and the results of the BEAST segmentation (Figure S4); dwelling time vs trend for equilibrium with *J*_*s*_ = −3.4 [*K*_B_*T*] (Figure S5); dwelling time vs trend for equilibrium with *J*_*s*_ = −3.6 [*K*_B_*T*] (Figure S6); histograms of the *T*_FAS_ data for equilibrium and nonequilibrium (Figure S7); impact of drive activation on assembly yield and time to first assembly for *M*_*T*_ = 3, *N* = 25 (Figure S8); impact of drive activation on assembly yield and time to first assembly for *M*_*T*_ = 4, *N* = 25 (Figure S9); impact of drive activation on assembly yield and time to first assembly for *M*_*T*_ = 2, *N* = 36 (Figure S10); board snapshots before and after drive activation (Figure S11) (PDF)

## Supplementary Material

ci4c02366_si_001.avi

ci4c02366_si_002.avi

ci4c02366_si_003.avi

ci4c02366_si_004.avi
