# Supplementary material for: Nonequilibrium Self-Assembly Control by the Stochastic Landscape Method
Source: J Chem Inf Model. 2025 Apr 8;65(8):4067–80. doi: 10.1021/acs.jcim.4c02366 (PMC12042269; doi:10.1021/acs.jcim.4c02366)
Supplement: Supplementary file 5 — ci4c02366_si_005.pdf [file ci4c02366_si_005.pdf]

Supporting Information: Nonequilibrium Self-Assembly Control by the Stochastic Landscape Method

Michael Faran† and Gili Bisker*,†,‡,¶,§,‖

†Department of Biomedical Engineering, Faculty of Engineering, Tel Aviv University, Tel Aviv 69978, Israel

‡The Center for Physics and Chemistry of Living Systems, Tel Aviv University, Tel Aviv 6997801, Israel

¶The Center for Nanoscience and Nanotechnology, Tel Aviv University, Tel Aviv 6997801, Israel

§The Center for Light-Matter Interaction, Tel Aviv University, Tel Aviv 6997801, Israel

‖The Center for Computational Molecular and Materials Science, Tel Aviv University, Tel Aviv 6997801, Israel

E-mail: bisker@tauex.tau.ac.il

Rate Constant Calculation

The characteristic timescale for a protein to diffuse its own size within a cellular environment can be estimated using the mean squared displacement of a Brownian particle and typical cellular parameters.

For a typical globular protein with a radius of gyration of approximately 2 nm,[1] the diffusion coefficient in cytoplasm is around 10 μm^2/s, which is about ten times slower than its diffusion rate in water.[2] Using the relation between time, displacement, and diffusion coefficient for a Brownian particle, given by $\tau \approx x^2/D$,[3] where x is the diffusion distance and D is the diffusion coefficient, we can estimate the time for a protein to diffuse its own diameter (approximately 4 nm) as: $\tau \approx (4\text{nm})^2/(10\mu\text{m}^2/\text{s}) \approx 4 \times 10^{-7}$s or 0.4 μs. The corresponding rate constant r_0, defined as the inverse of this timescale, is therefore: $r_0 \approx 1/\tau \approx 2.5 \times 10^6, \text{s}^{-1}$.

This rate represents the typical frequency at which a protein moves a distance equal to its own size in a cellular environment, providing a relevant timescale for protein-protein interactions and molecular recognition processes within cells.[4]

Additional Equilibrium Simulation Results

This section presents the equilibrium simulation results, including self-assembly yield and time to first assembly as functions of the strong energy parameter J_s. Additionally, examples of total energy trajectories segmented and analyzed for trends over multiple simulation realizations are provided, as detailed in the main text.

Fig. S1 presents the same data shown in Fig. 2 of the main text, but without normalizing the T_{FAS} values. In addition, the single data point treating all unassembled trajectories as their maximum possible T_{FAS} value (as was conducted for $\hat{T}_{FAS}$ in Fig. 2), is shifted into their mean T_{FAS} value instead. The three distinct self-assembly regions, as described in the main text, are also clearly visible in this representation.

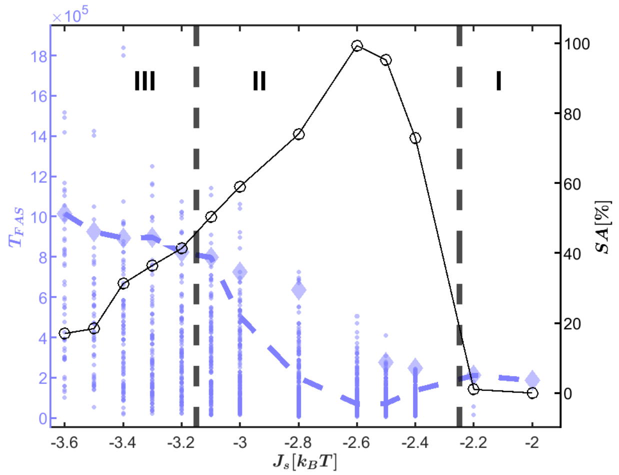

Figure S1: Equilibrium simulation results for the parameters in Table 1, with $M_T = 2$, without the normalization as conducted in the main text. The y-axis shows T_{FAS} distribution versus J_s, based on 288 simulation realizations. The dashed purple line represents the median $T_{FAS}(J_s)$. Non-assembled results are collectively represented as diamonds, appearing in their mean T_{FAS} values. The black line corresponds to the right y-axis. It shows the assembly percentage versus J_s. Dashed vertical black lines qualitatively separate the three self-assembly regions (I-III) discussed in the main text.

Fig. S2 presents the normalized self-assembly time versus the strong binding energy parameter for systems with three and four stored targets, each consisting of 25 particles. In contrast, Fig. S3 displays the corresponding curve for a system with two stored targets and 36 particles. As was discussed in the main text, for the system with two targets and 25 particles, the median time to first assembly $T_{FAS}(J_s)$, normalized by the maximum result for each J_s value, shows a plateau, followed by a concave region, and then another plateau, highlighting three distinct self-assembly dynamic regimes.

Fig. S4 provides examples of the data used to construct Fig. 3 from the main text, highlighting the observed inverse relationship between trend and dwelling times. Careful examination of these segments and their corresponding trend values further supports this inverse relationship.

Fig. S5 and Fig. S6 show the macro-state dwelling times mapped against the trend value for $J_s = -3.4$ $[k_BT]$ and $J_s = -3.6$ $[k_BT]$, respectively. These maps were constructed during the learning phase of the closed-loop algorithm under equilibrium conditions.

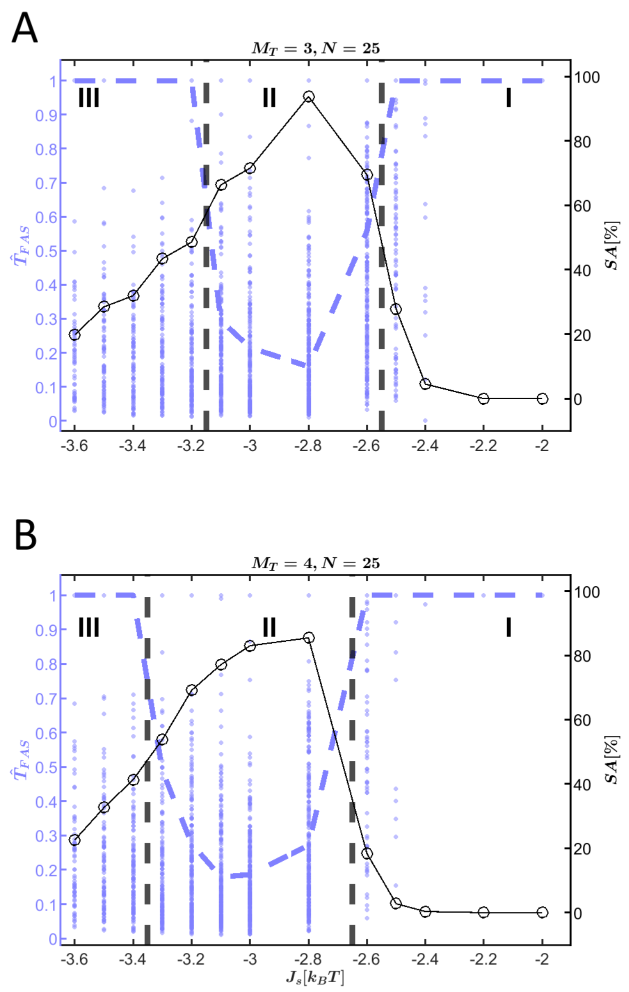

Figure S2: Equilibrium simulation results for the parameters in Table 1, with (A) $M_T = 3$ and (B) $M_T = 4$. The left y-axis shows the normalized $\hat{T}_{FAS}$ distribution versus J_s, based on 288 simulation realizations, where $\hat{T}_{FAS}$ is the assembly time, T_{FAS}, normalized by the maximum simulation time per J_s. The dashed purple line represents the median $\hat{T}_{FAS}(J_s)$. Pale purple dots indicate values where assemblies occurred within the simulation time frame. Non-assembled results are grouped at $\hat{T}_{FAS} = 1$. The black line corresponds to the right y-axis. It shows the assembly percentage versus J_s. Dashed vertical black lines qualitatively separate the three self-assembly regions (I-III) discussed in the text.

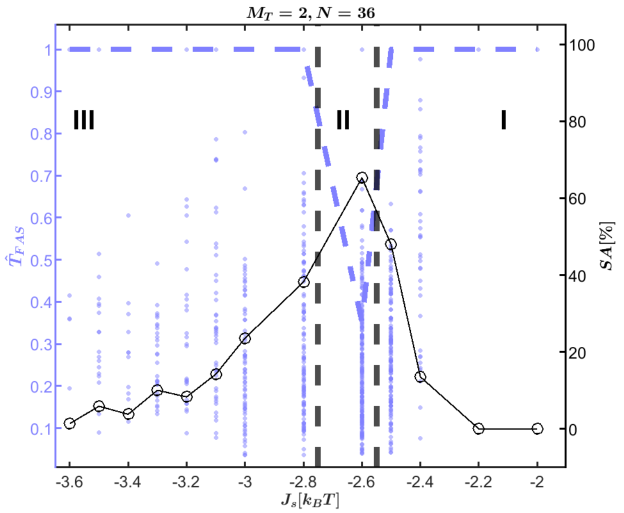

Figure S3: Equilibrium simulation results for the parameters in Table 1, with $M_T = 2$ and $N = 36$ particles. The left y-axis shows the normalized $\hat{T}_{FAS}$ distribution versus J_s, based on 288 simulation realizations, where $\hat{T}_{FAS}$ is the assembly time, T_{FAS}, normalized by the maximum simulation time per J_s. The dashed purple line represents the median $\hat{T}_{FAS}(J_s)$. Pale purple dots indicate values where assemblies occurred within the simulation time frame. Non-assembled results are grouped at $\hat{T}_{FAS} = 1$. The black line corresponds to the right y-axis. It shows the assembly percentage versus J_s. Dashed vertical black lines qualitatively separate the three self-assembly regions (I-III) discussed in the text.

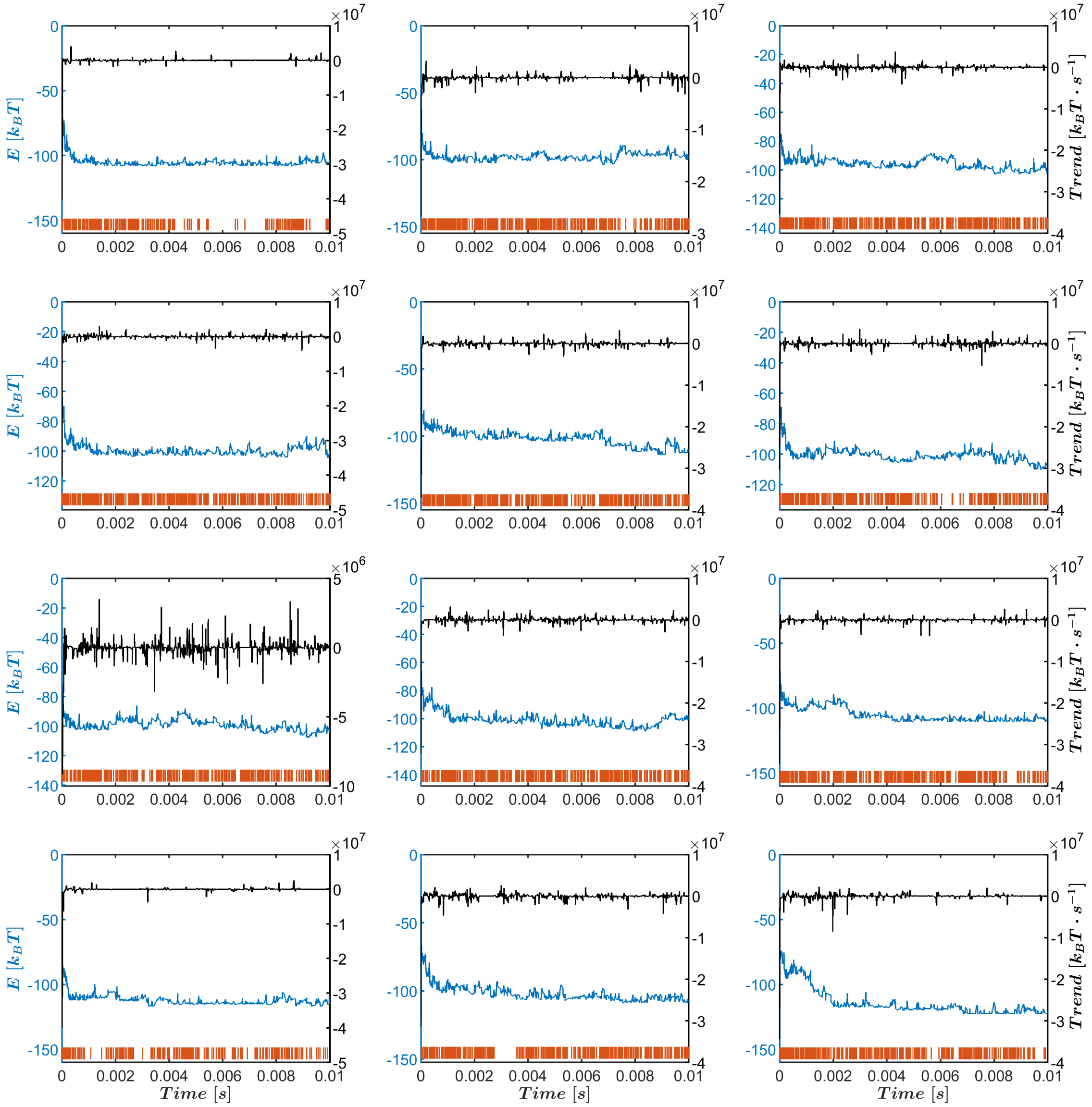

Figure S4: Energy versus time trajectories for 12 simulation realizations with default parameters and $J_s = -3.5[k_BT]$ are shown. The energy trajectories are displayed in blue, while their corresponding trend over time is represented in black. Consecutive orange vertical lines mark segments identified using the stochastic landscape method through the BEAST algorithm.

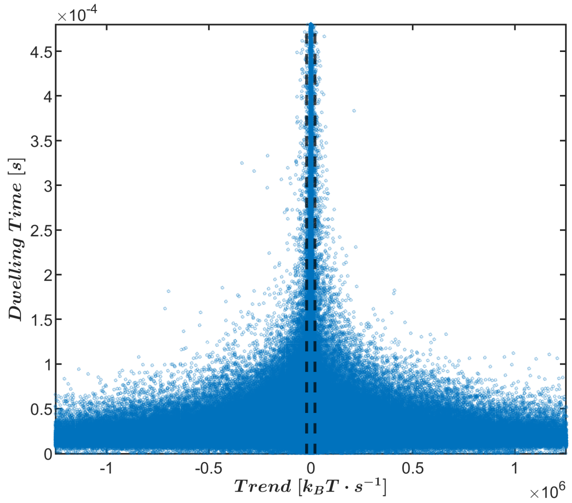

Figure S5: The dwelling time versus trend of the overall stochastic landscape segments obtained using the BEAST algorithm in almost real-time for $J_s = -3.4[k_B T]$. The rest of the simulation physical parameters follow Table 1 of the main text. The kinetic trap region (T^*), centered around trend zero, defines the conditions for drive activation.

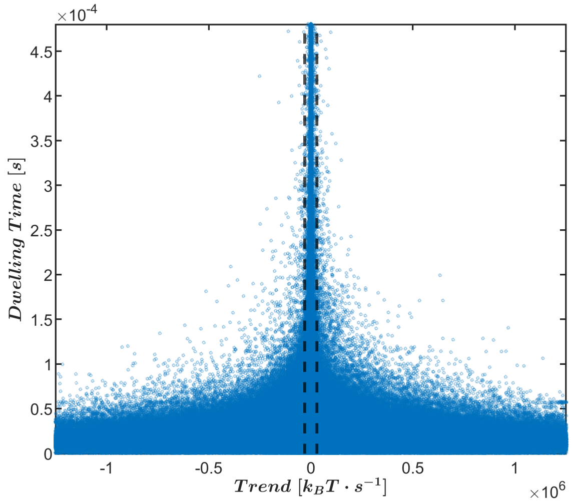

Figure S6: The dwelling time versus trend of the overall stochastic landscape segments obtained using the BEAST algorithm in almost real-time for $J_s = -3.6[k_B T]$. The rest of the simulation physical parameters follow Table 1 of the main text. The kinetic trap region (T^*), centered around trend zero, defines the conditions for drive activation.

Additional Nonequilibrium Simulation Results

This section presents the results of the nonequilibrium drive shock activation, following the methodology described in the main text (see Fig. 3). Fig. S7 illustrates the distribution of T_{FAS} through histograms for both equilibrium and nonequilibrium simulations, showing the optimized drive for maximizing assembly yield across different values of J_s. Data for each histogram were collected from 48 simulation realizations with consistent parameters.

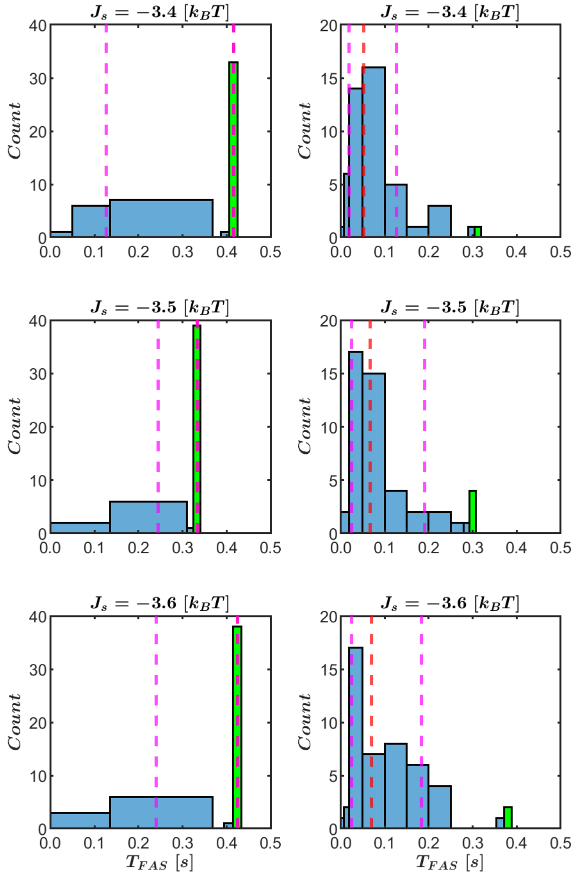

Figure S7: The T_{FAS} histograms for equilibrium conditions (left column) and nonequilibrium shock activation with an amplitude of $\rho = 1.5$ (right column) are displayed, for (A) $J_s = -3.4$ $[k_BT]$, (B) $J_s = -3.5$ $[k_BT]$, and (C) $J_s = -3.6$ $[k_BT]$, respectively. The dashed red vertical lines indicate the median values of each histogram, while the dashed purple lines represent the 16th and 84th percentiles of the data. The green bin depicts realizations where no assembly occurred, aggregated altogether.

To accurately represent the underlying data distribution, we employed unequal binning: equal binning on a logarithmic scale was used for values below the median. At the same time, standard Scott's rule[5] was applied for values above the median. Additionally, all instances where assembly did not occur were grouped into a single bin for clarity.

A visual comparison of the histograms reveals that iterative shock activation substantially improves assembly yield and reduces the first assembly time. This is evident from the reduced count of non-assembly instances, represented by the last bin, and the positioning of the median lines in red for the nonequilibrium case at lower T_{FAS} values. In contrast, the median and 84th percentile lines coincide at the last bin for equilibrium conditions, indicating that more than half of the simulation realizations did not result in an assembly event.

Figs S8, S9 and S10 display the assembly yield data and the time to first assembly improvements for physical system configurations with three and four targets stored in memory using 25 particles, and two targets with 36 particles, respectively. Across all cases, when using shock amplitude values optimized to maximize assembly yield, a corresponding reduction in $\tilde{T}_{FAS}$ was observed compared to equilibrium conditions. The consistent convex pattern of assembly yield versus shock amplitude across these simulated systems demonstrates the robustness of the proposed method concerning varying system parameters.

For the optimal drive value of $\rho = 1.5$ in a system with two encoded targets and 25 particles (see main text), Fig. S11 shows typical board states before and after drive activation.

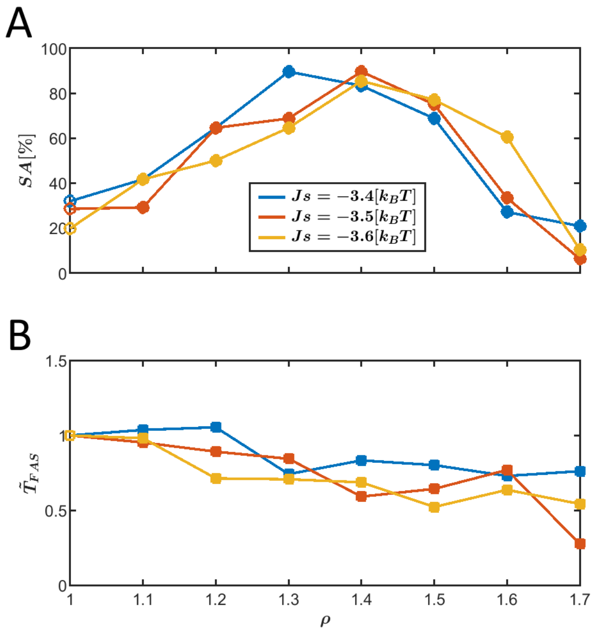

Figure S8: The impact of drive activation on the assembly yield ($SA[\%]$) and the rescaled mean time to first assembly ($\tilde{T}_{FAS}$) for $N = 25$ particles and $M_T = 3$ stored targets. (A) Assembly yield versus the drive amplitude ρ. (B) Mean time to first assembly, rescaled by the equilibrium mean time to first assembly, plotted against the drive amplitude ρ. Results are provided for $J_s = -3.4$, -3.5, and -3.6 $[k_BT]$. The mean time to first assembly and assembly yield are calculated based on 48 simulation realizations. The empty markers in $\rho = 1$ depict the equilibrium values.

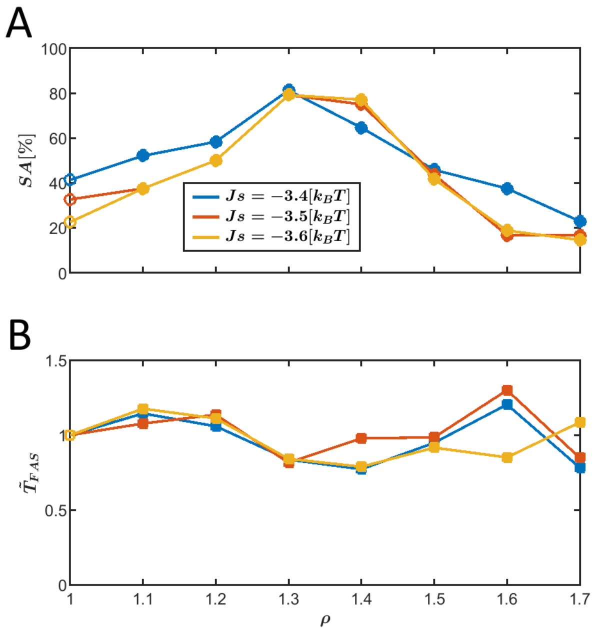

Figure S9: The impact of drive activation on the assembly yield ($SA[\%]$) and the rescaled mean time to first assembly ($\tilde{T}_{FAS}$) for $N = 25$ particles and $M_T = 4$ stored targets. (A) Assembly yield versus the drive amplitude ρ. (B) Mean time to first assembly, rescaled by the equilibrium mean time to first assembly, plotted against the drive amplitude ρ. Results are provided for $J_s = -3.4$, -3.5, and -3.6 $[k_BT]$. The mean time to first assembly and assembly yield are calculated based on 48 simulation realizations. The empty markers in $\rho = 1$ depict the equilibrium values.

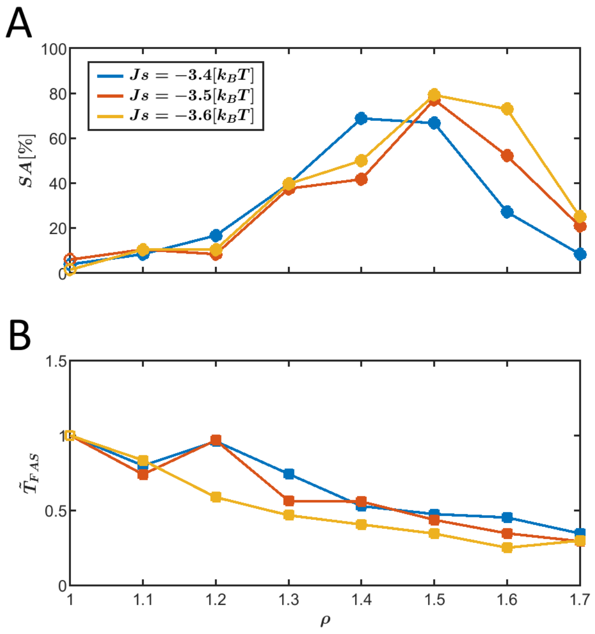

Figure S10: The impact of drive activation on the assembly yield ($SA[\%]$) and the rescaled mean time to first assembly ($\tilde{T}_{FAS}$) for $N = 36$ particles and $M_T = 2$ stored targets. (A) Assembly yield versus the drive amplitude ρ. (B) Mean time to first assembly, rescaled by the equilibrium mean time to first assembly, plotted against the drive amplitude ρ. Results are provided for $J_s = -3.4$, -3.5, and -3.6 $[k_BT]$. The mean time to first assembly and assembly yield are calculated based on 48 simulation realizations. The empty markers in $\rho = 1$ depict the equilibrium values.

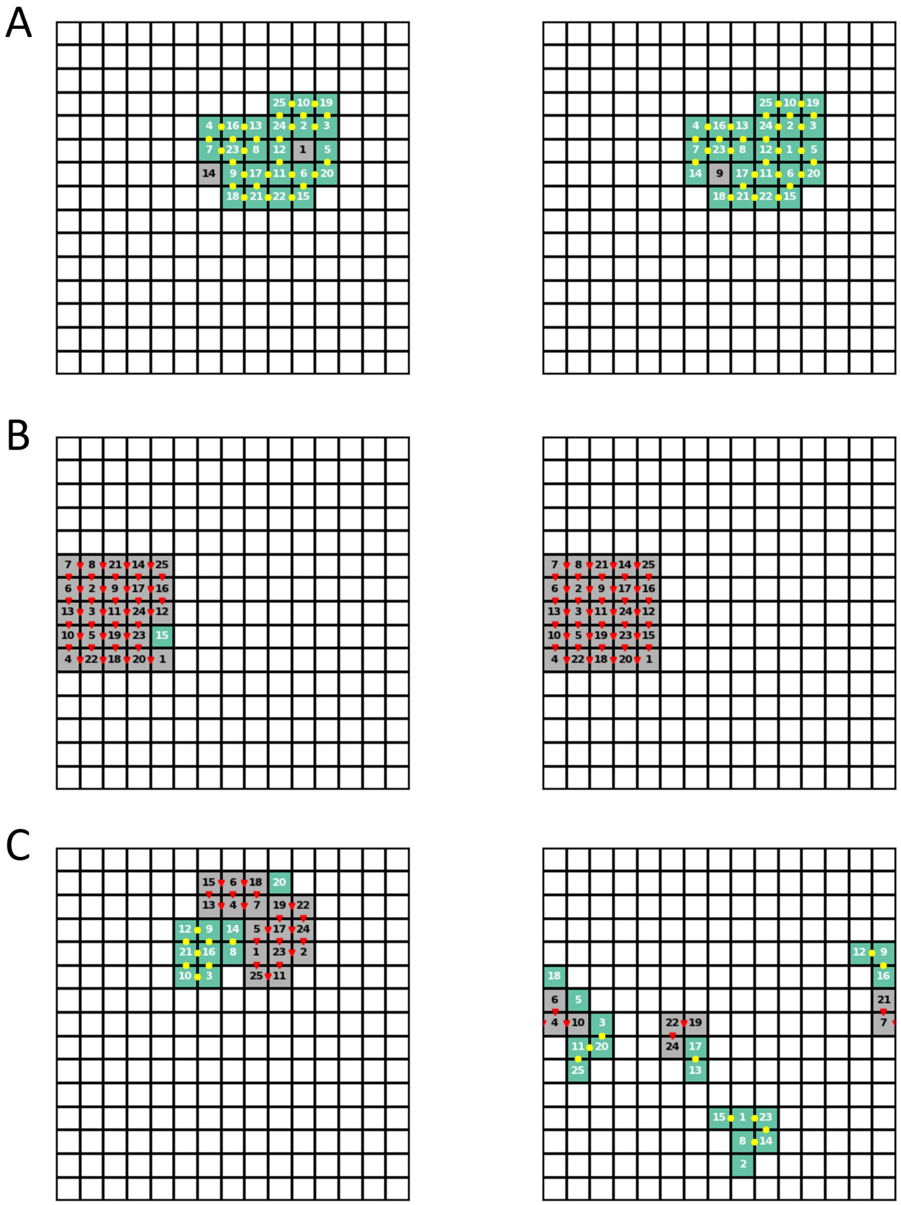

Figure S11: Typical board state screenshots, captured before and after shock activation, for different shock amplitudes are shown. Rows A, B, and C display the board states before (left column) and after (right column) shock activation for drives of $\rho = 1.1$, 1.4, and 1.7, respectively.

References

(1) Smilgies, D.-M.; Folta-Stogniew, E. Molecular weight–gyration radius relation of globular proteins: a comparison of light scattering, small-angle X-ray scattering and structure-based data. *J. App. Cryst.* **2015**, *48*, 1604–1606.

(2) Dix, J. A.; Verkman, A. Crowding effects on diffusion in solutions and cells. *Ann. Rev. Biophys.* **2008**, *37*, 247–263.

(3) Metzler, R.; Klafter, J. The random walk's guide to anomalous diffusion: a fractional dynamics approach. *Phys. Rep.* **2000**, *339*, 1–77.

(4) Elowitz, M. B.; Surette, M. G.; Wolf, P.-E.; Stock, J. B.; Leibler, S. Protein mobility in the cytoplasm of Escherichia coli. *J. Bact.* **1999**, *181*, 197–203.

(5) Knuth, K. H. Optimal data-based binning for histograms. *arXiv preprint physics/0605197* **2006**, `https://arxiv.org/abs/physics/0605197` (accessed 2024-12-05).
